# Immobilization of pH-sensitive CdTe Quantum Dots in a Poly(acrylate) Hydrogel for Microfluidic Applications

**DOI:** 10.1186/s11671-017-2069-x

**Published:** 2017-04-27

**Authors:** M. Franke, S. Leubner, A. Dubavik, A. George, T. Savchenko, C. Pini, P. Frank, D. Melnikau, Y. Rakovich, N. Gaponik, A. Eychmüller, A. Richter

**Affiliations:** 1 Institute of Semiconductors and Microsystems, Faculty of Electrical and Computer Engineering, Technische Universität Dresden, 01062 Dresden, Germany; 2 Physical Chemistry, Technische Universität Dresden, 01062 Dresden, Germany; 3 Center for Advancing Electronics Dresden (cfaed), Technische Universität Dresden, 01062 Dresden, Germany; 4 Centro de Física de Materiales (MPC, CSIS- UPV/EHU), Paseo Manuel de Lardizabal 5, Donostia-San Sebastián, 20018 Spain; 5 Donostia International Physics Center (DIPC), Paseo Manuel de Lardizabal 5, Donostia-San Sebastián, 20018 Spain; 6 IKERBASQUE, Basque Fondation for Science, Alameda Urquijo 365, Bilbao, 48011 Spain; 7 CIC nanoGUNE Consolider, Tolosa Hiribidea 76, Donostia San Sebastian, 20018 Spain; 8 ITMO University, 197101 Kronverksky prospect, 49, Saint Petersburg, Russia; 9 Indian Institute of Science Education and Research (IISER), Thiruvananthapuram, Kerala India

**Keywords:** Hybrid material, PH-sensitive polymer, Quantum dots, Cadmium telluride, Poly(acrylate) hydrogel, Microfluidic valve, Photoluminescence detection

## Abstract

Microfluidic devices present the basis of modern life sciences and chemical information processing. To control the flow and to allow optical readout, a reliable sensor material that can be easily utilized for microfluidic systems is in demand. Here, we present a new optical readout system for pH sensing based on pH sensitive, photoluminescent glutathione capped cadmium telluride quantum dots that are covalently immobilized in a poly(acrylate) hydrogel. For an applicable pH sensing the generated hybrid material is integrated in a microfluidic sensor chip setup. The hybrid material not only allows in situ readout, but also possesses valve properties due to the swelling behavior of the poly(acrylate) hydrogel. In this work, the swelling property of the hybrid material is utilized in a microfluidic valve seat, where a valve opening process is demonstrated by a fluid flow change and in situ monitored by photoluminescence quenching. This discrete photoluminescence detection (ON/OFF) of the fluid flow change (OFF/ON) enables upcoming chemical information processing.

## Background

Microfluidic systems enable an efficient way to analyze chemical, biological and reaction parameters and thereby present the basis of many modern analysis and information processing platforms yet. Nowadays, a huge number of chemical reactions can be performed simultaneously on small-scaled microfluidic devices. This allows to reduce processing time, the consumption of energy and chemicals, and may also improve the reliability of the process [1]. In microfluidic research, the concept of active fluid flow manipulation and processing by integrated circuits, the so-called labs on a chip, is of core importance. This technology shows similar properties of manipulating streams as those existing in electron based microelectronic information technology [2–9]. Beyond MEMS-based microfluidics, approaches with active fluid flow manipulation are attractive to monitor interaction of active material components with thermodynamic carriers of information such as changes in temperature, substance concentration, or pH value.

In recent years, strong efforts were made in the field of “smart” hydrogels in order to employ them as active elements in microfluidic platforms/devices [9–16]. These active elements are based on crosslinked polymer networks in aqueous solutions showing a stimuli-induced volume-phase transition (VPT) between a swollen and shrunken state which can be characterized by drastic change in the swelling degree. Here, two major groups of polymeric hydrogels exist for realization of smart application. Stimuli-sensitive hydrogels, e.g., many poly(acrylamide) representatives, can be in a critical swelling equilibrium depending on the free enthalpy of mixing polymer network and water as a swelling agent and the elasticity of polymer chains. The VPT occurs at even small changes of environmental parameters like temperature, salts, other solvents and many more [13, 17]. Polyelectrolyte hydrogels can be stimulated to swell or shrink by pH changes. In this case, the VPT is triggered by ionic repulsion forces induced by charging of weak acidic or basic groups inside the gel with mobile counter ions, like hydronium or hydroxide ions [13]. Hence, the VPT occurs near the pK_a_ or pK_b_ value of the polyelectrolyte hydrogel. Polyelectrolyte hydrogels for smart application are made of copolymers from poly(acrylic acid) and poly(2-hydroxyethyl methacrylate) [10], poly(vinyl alcohol) and poly(acrylic acid) [13] or poly(acrylate) (PA) [9] and show huge swelling degrees.

Importantly, a permanent readout of the current status of active microfluidic elements is highly demanded since this will serve as an interface with current electronic semiconductor technologies and at the same time will enable the collection of information about the dynamics and kinetics of chemical processes [18]. One possible way to interface electronics with the microfluidic domain is given by the implementation of an optical detection system directly in the microfluidic channel. Due to their unique electrochemical or optoelectronic properties, nanoparticles (NPs) made of metallic [19, 20] or semiconductive [21] materials are often applied for sensing application in microfluidic systems. However, many of these NP based systems suffer from leaching and instability of the NPs. In this regard, the chemical immobilization of NPs on surfaces or in porous matrices is a known alternative to overcome these problems [22–24]. In case of soft-matter, active material components, especially hydrogels, it is obvious to use these as host matrix for NPs. The polymers facilitate compatibility with the NPs, a stable immobilization and provide desired optical properties. These requirements are mandatory for a suitable on-chip sensor. Many examples of these hybrid materials were reported to date [24–33] prepared by different coupling methods such as grafting the polymer to the NP ligand shell [26, 27, 33] or capping the NP by the polymer [31, 32, 34]. Apart from these hybrid materials, optically active quantum dots (QDs) can be considered as a promising material for the functionalization of the hydrogels, as the optical readout signal (which is the strong and photostable emission of QDs), is extremely sensitive to pH changes [35, 36]. Hence, QDs are applied as pH sensors and have been used in the development of bio- and ratiometric sensing platforms [37–41]. However, none of the realized sensing approaches show a permanently immobilization of QDs in a microfluidic chip system. In the study at hand, we developed a new hybrid material that consists of pH sensitive, fluorescent QDs made of glutathione (Glu) capped cadmium telluride (CdTe) which are immobilized by covalent attachment in a polyelectrolyte PA hydrogel. The QDs based hybrid system was further integrated in microfluidic chips in order to demonstrate a successful combination of an optical pH sensor and an active valve element.

## Methods

### Chemicals

All chemicals used were of analytical grade or of the highest purity available and used as received: L-Glutathione reduced (Glu, Aldrich), Cadmium (II) perchlorate hexahydrate (Cd(ClO_4_)_2_•6H_2_O, Alfa Aesar), Aluminum telluride (Al_2_Te_3_, Cerac Inc.), Sodium hydroxide (NaOH, Aldrich), Sulfuric acid (H_2_SO_4_, Aldrich), Sodium acrylate (NaA, Aldrich), *N*,*N*’-Methylenebis(acrylamide) (Bis, Aldrich), 2-Hydroxy-4′-(2-hydroxyethoxy)-2-methylpropiophenone (PI, Photoinitiator, Aldrich), *N*-Hydroxysuccinimide (NHS, Aldrich), *N*-Ethyl-*N’*-(3-dimethylaminopropyl)carbodiimide (EDC, Aldrich), buffer pH 2.0 (Citric acid, sodium hydroxide, hydrogen chloride solution, Certipur®, Merck), buffer pH 4.0 (Citric acid, sodium hydroxide, hydrogen chloride solution, Certipur®, Merck), buffer pH 5.5 (Citric acid, sodium hydroxide solution, Certipur®, Merck) buffer pH 7.0 (Disodium hydrogen phosphate, potassium dihydrogen phosphate solution, Certipur®, Merck), buffer pH 9.0 (Boric acid, potassium chloride, sodium hydroxide solution, Certipur®, Merck), buffer pH 10.0 (Boric acid, potassium chloride, sodium hydroxide solution, Certipur®, Merck), buffer pH 11.0 (Boric acid, potassium chloride, sodium hydroxide solution, Certipur®, Merck), poly(dimethylsiloxane) (PDMS, Sylgard 184, 10:1 mixture, Dow Corning). For all reactions and cleaning steps ultrapure water was used.

### Synthesis of Glu-Capped CdTe QDs

The Glu capped CdTe QDs were synthesized according to a modified, previously reported procedure [42]. In short, 2.75 mmol of Cd(ClO_4_)_2_•6H_2_O were dissolved in 125 mL water and followed by adding of 3.6 mmol Glu as the stabilizer. The pH of the solution was adjusted to 12 by dropwise adding of 1 M NaOH. The resulting solution was placed in a three-necked flask and flushed with Argon for 30 min to remove all oxygen. The gaseous Te-precursor hydrogen telluride H_2_Te was generated by reacting 0.2 g (0.458 mmol) Al_2_Te_3_ with 5 mL of a 0.5 M H_2_SO_4_ solution and passed through the solution under slow argon flow and vigorous stirring. The growth of the CdTe QDs proceeded under open air conditions at 100 °C and reflux. After precipitation in isopropanol and re-dispersing in water, the final colloidal QDs solution was obtained.

### Synthesis of PA Hydrogel

The macroscopic PA hydrogel was synthesized and patterned by a photopolymerization procedure [9, 16]. 17.5 mmol of NaA, 0.27 mmol Bis, and 0.18 mmol PI were weighted out and filled in a 50 mL round flask with septum followed by flushing with argon to remove all oxygen and adding of 14 mL degassed water. The reaction mixture was stirred under inert atmosphere and kept away from light until receiving a clear solution. For the fabrication of the PA hydrogel a 20 × 20mm black poly(ethylene terephthalate) (PET) mold with a depth of 140 μm was filled with the reaction mixture under argon atmosphere and sealed with a 1 mm thick ozone activated transparent PET substrate. Photopolymerization and patterning were performed by exposing the reaction mixture to UV-light through a photo mask (1-mm square structures, TypoPhot TO-G, plotted by a Herkules Imagesetter with 5080 dots/inch) with a UV-Lamp (Mercury/xenon short arc lamp, 1000 W, Lot Oriel, wavelength of maximum emission 365 nm, power 16 mW/cm^2^). The resulting PA hydrogel squares were immersed for cleaning in water over night while sticking to the transparent PET substrate.

### Synthesis of CdTe QDs PA Hydrogel Hybrid Material

For the coupling of the CdTe QDs with the PA hydrogel a modified post-polymerization procedure with EDC/NHS-mediated amide coupling was applied [26, 27]. PA hydrogel onto the PET was firstly immersed in a 0.1 M aqueous solution of EDC and secondly in a 0.1 M aqueous solution of NHS each for 1 h and interrupted by 1 h cleaning in water. After further cleaning with water the PA hydrogel with activated NHS ester groups was fully covered with pure CdTe QD solution and immersed for 24 h. After purification in water, the PA hydrogel CdTe hybrid material was separated from PET substrate by a razor blade and dried at room temperature.

### Microfluidic Chip Fabrication

The fabrication of microfluidic sensor chip and valve structure was performed by a standardized soft lithographic procedure [14, 16]. Both molding masters consisted of a structured double-layer dry-film-resist (Ordyl SY355, Elga Europe) resulting in a channel height of about 100 μm on a glass slide. An A10:B1 mixture of PDMS was prepared and casted over the molding masters (PDMS thickness 5 mm). After degassing, the PDMS was cured at 60 °C for 24 h. Microfluidic chips were assembled after punching the fluidic contacts by pick-and-place-transfer the CdTe QD PA hybrid material to the anticipated cage structure of the chip and sealed by close clamping with a glass slide on top.

### Characterization and Methods

UV/Vis Spectra were recorded using a UV-Vis spectrophotometer Carry 5000 (Varian Inc., USA) and fluorescence spectra were measured using a FluoroMax 4 spectrofluorimeter (Instruments SA, USA). The CdTe QDs PA hydrogel hybrid material on the PET substrate was immersed in water or the appropriate buffer for 1 h and immediately examined by direct placing in the light path.

The time resolved confocal fluorescence lifetime imaging microscopic (FLIM) investigations of the hybrid material were performed using the MicroTime 200 (PicoQuant, Germany) while flushing the different buffer solution (pH 7.0, 9.0, 11.0) with a tube and syringe manually through the microfluidic channel. The samples were excited by a picosecond laser emitting at 485 nm. FLIM images were obtained by PL decay mapping (40 × 40 μm) when each pixel in the FLIM image gives the lifetime at a particular position in area, while monitoring the entire photoluminescence (PL) spectrum.

The pH sensing with the microfluidic sensor setup was carried out with the fluorescence microscope Axiostar (Zeiss, Germany) which was connected to a fiber glass spectrometer (OceanOptics, USA). For alternating the pH value 5.5 and 10.0 the microfluidic chip was connected with a Y junction to two syringe pumps La 100 (Landgraf Laborsysteme, Germany) alternately running with a flow rate of about 100 μl/min. Each cycle lasted 30 min. Excitation of the CdTe QD PA hybrid material was carried out at λ_ex_ = 450 nm and the highest emission in each cycle was depicted. For better comparison, the highest emission intensities were normalized.

The valve properties were carried out with the microfluidic valve setup and the macroscope M450 (Wild Heerbrugg, Switzerland). The PL images were taken under excitation by a UV-lamp. For changing the pH value from 10.0 to 5.5 the microfluidic chip was connected with a Y junction to two pressure pumps (AF-1, Elveflow, France) while just one was running. The flow rate was determined by a flow sensor (SLI-1000, Sensirion AG, Switzerland) placed behind the chip outlet. All taken PL images were corrected for contrast and brightness by RawTherapee software.

## Results and Discussion

**Fig. 1:**
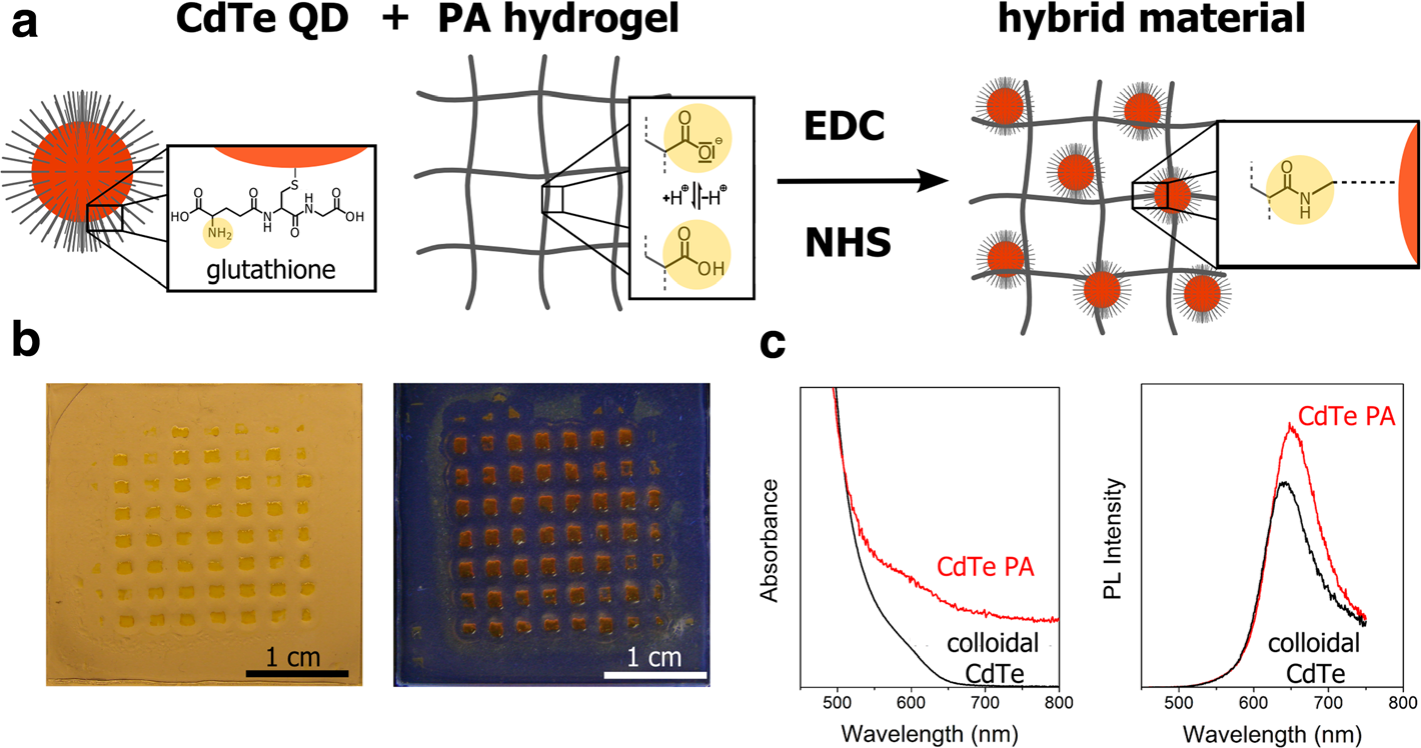
The immobilization of Glu capped CdTe QDs within PA hydrogels applying EDC/NHS-mediated coupling and hybrid material formation (**a**) yield homogeneously colored and emitting hydrogels as displayed under white light (**b** *left*) and UV-light (**b** *right*). The absorption (**c** *left*) and PL emission spectra (**c** *right*) show preserved optical features of the QDs in the hydrogel

A successful immobilization of QDs within a PA hydrogel requires a suitable covalent or physical coupling. In this regard, a new Glu capped CdTe QD material was synthesized according to previous reported procedures of thiol capped CdTe QD [42]. The introduced amino groups of the Glu stabilizer molecules are utilized for covalently EDC/NHS-mediated amide coupling to the carboxylic groups of the PA hydrogel, as schematically depicted in Fig. 1a. The resulting CdTe QD PA hydrogel hybrid material shows a pronounced color, which is typical for the CdTe QDs used, see Fig. 1b. The absorption and PL spectra, shown in Fig. 1c, underline the preservation of the emission properties of colloidal QDs features in the hybrid material even after several washing steps with water. Only a weak shift of PL band in the spectrum was observed.

**Fig. 2:**
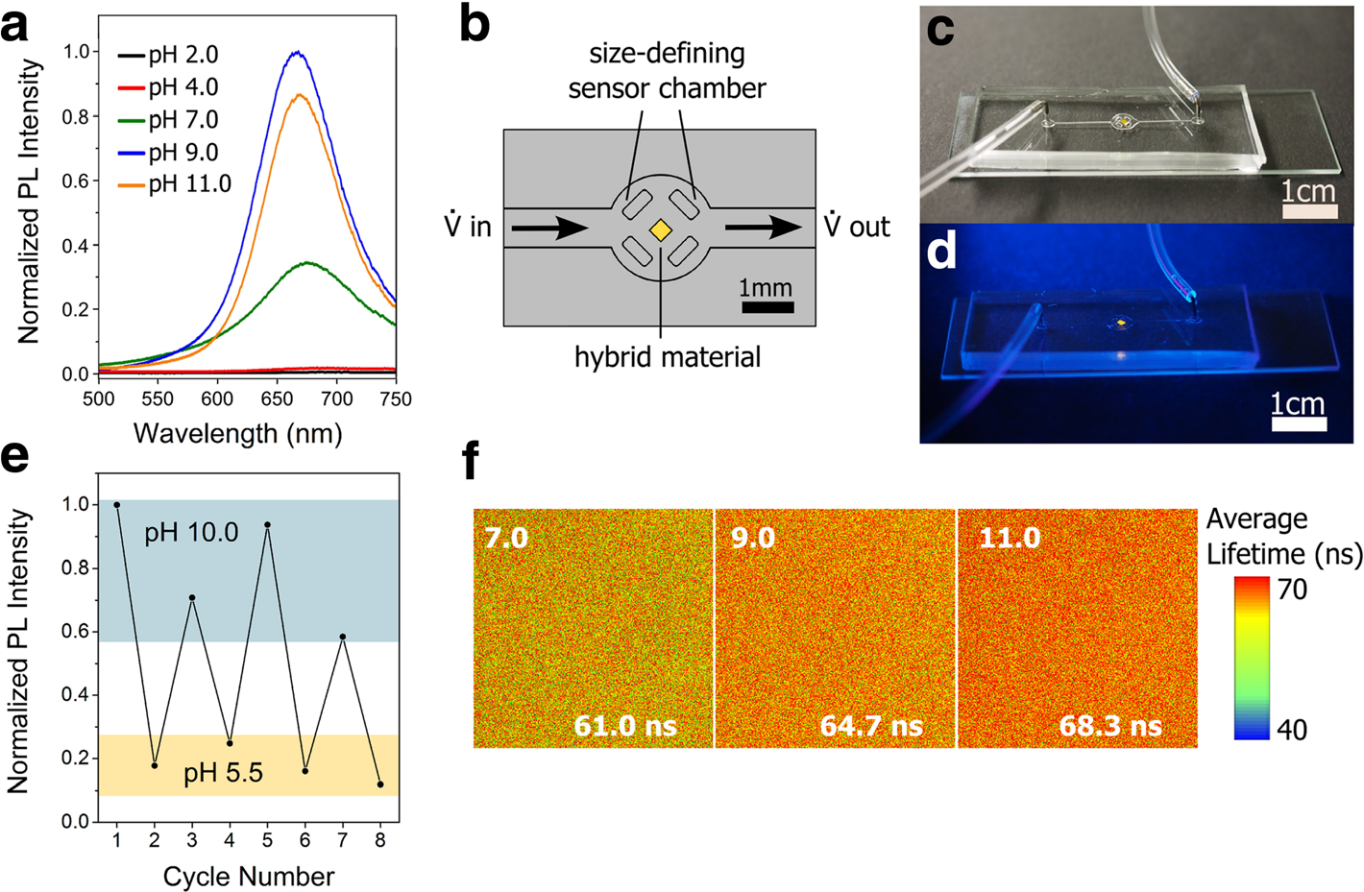
Pronounced pH dependency of PL intensity of CdTe QDs immobilized within the PA hydrogel, λ_ex_ = 450 nm (**a**), schematic sensor chamber of microfluidic setup (top view) (**b**) and photo of built setup taken under white light (**c**) and UV-light (**d**). Large pH deviations sensed by the PL intensity when alternately changing the buffer from pH 10.0 to 5.5 (8 cycles) (**e**). pH dependent PL changes measured by FLIM of CdTe-PA gel in buffers with pH 7.0, 9.0 and 11.0, respectively (**f**)

The CdTe QD PA hydrogel hybrid material exhibits a pH dependent PL that was spectroscopically monitored as shown in Fig. 2a. While the position of the emission maximum only slightly shifts when changing pH from 9.0 (λ_em_ = 669 nm) to 4.0 (λ_em_ = 685 nm), the PL intensity shows an initial improvement of PL efficiency (as pH changes from 11.0 to 9.0) followed by a pronounced reduction of PL intensity upon decreasing the pH from 9.0 to 2.0. Various studies clarified the effect of pH on QDs PL and stability of water-soluble cadmium chalcogenide QDs [35, 36, 43–48]. Our previous work revealed the complexity and dynamics of the ligand shell of cadmium chalcogenide QDs synthesized in water [35, 49, 50]. It was conceived that the effect of the pH is caused by structural changes of the ligand shell, interaction between charged ligands and the electronic structure of the core, dissociation of ligands from the surface due to protonation, counter ion effects, the additional coordination of cadmium to carboxyl groups and, what is most likely, by the sum of different contributions. A comprehensive study on the pH-dependent PL of various thiol capped CdTe QDs was made by Xu et al. [36]. It summarized several phenomena and revealed the pK_a_ value of terminal groups (e.g., carboxylic group for thioglycolic acid, amino group for 2-mercaptoethylamine) of the applied thiol ligand which mainly influences the optimal pH value [36]. Considering the more complex structure of the Glu stabilizer, a sole effect of a single functional group is not possible. However, the huge number of carboxylic groups from the Glu and the PA hydrogel most likely affect the pH dependence of PL in the dominant way. Decreasing the pH from basic solutions (pH 11.0) to pH 9.0 leads to a reduction of electrostatic repulsion between free and bound ligand. Consequently, more free ligands can diffuse into the QD ligand shell and thereby improve the PL efficiency. Further decreasing of the pH to 7.0, 4.0, and 2.0 reduces the electrostatic repulsion of the QDs itself which leads to aggregation and loss of stability (Fig. 2a). Having acidic condition at pH 2.0 and 4.0, the PL intensity is totally and non-reversibly quenched, due to aggregation and QDs surface oxidation.

The intrinsic pH sensitivity of QDs is advantageous and contributes to a high sensitivity. This allows the distinction between acidic and basic solution simply by monitoring the PL intensity.

For realizing an active component for pH sensing, we integrated the CdTe QDs with PA hydrogel hybrid material into a microfluidic system. The setup is illustrated schematically in Fig. 2b and in real life in Fig. 2c, d. The structured QDs hydrogel was placed in a micro-structured cage. The cage setup allowed the integration of the hydrogel whilst enabling a continuous perfusion flow even when the hydrogel was fully swollen. The solution respectively the pH value in the channel was changed from pH 5.5 to pH 10.0 for 8 cycles by varying the buffer solution. The change in pH evoked an alteration of the PL intensity accordingly as shown in Fig. 2e. The measurements of the PL intensity provide a mean and clear distinction between both buffers used even after several cycles. This behavior can be applied for pH sensor application in microfluidic devices even when the material is totally swollen.

Moreover, for investigating pH changes, a detection of the fluorescence lifetime is favorable. A big advantage of using fluorescence lifetime analysis is the fact that this method does not depend on concentration, absorption by the sample, photo-bleaching and/or excitation intensity and therefore it is more robust than intensity based methods. At the same time, the fluorescence lifetime depends on a number of environmental parameters such as pH, ion or oxygen concentration, which makes this technique very useful for sensing applications [51]. In our experiments, the hybrid material was placed in different buffer solutions with pH values of 7.0, 9.0, and 11.0. Measured PL lifetime maps (Fig. 2f) clearly show the increase in PL lifetime with increasing pH (changes of color of the FLIM images from greenish yellow for pH = 7.0 to dark orange for pH = 11.0). To characterize this dependence quantitatively, we employed average (over each FLIM image) lifetimes calculated using an approach described in [52]. As indicated in Fig. 2f, the average PL lifetimes of the QD hydrogel hybrid composite increase from 61.0 to 68.3 ns when the solution is changed from neutral to basic pH value.

**Fig. 3:**
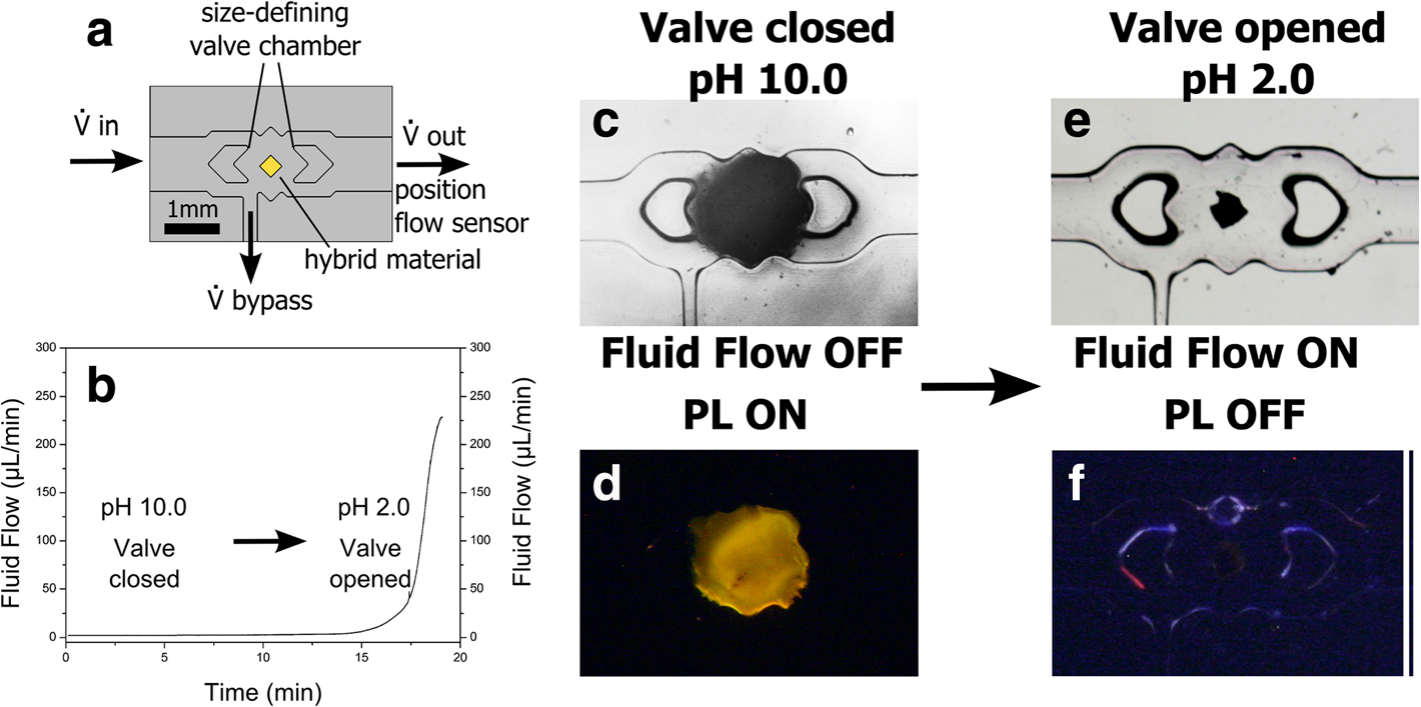
Schematic of the swelling behavior of the CdTe QD PA hydrogel hybrid material utilized in a microfluidic valve (**a**). Increase in fluid flow as a result of changing pH from 10 to 2 (**b**). Closed valve with swollen and photoluminescent hybrid material under white light (**c**) and UV-light (**d**). Opened valve with shrunken and non-photoluminescent hybrid material under day light (**e**) and UV-light (**f**)

The hybrid material shows a high sensitivity in the full pH range apart from the VPT of the polyelectrolyte PA hydrogel as described above. PA hydrogel undergo a VPT triggered by pH variation near the pK_a_ value of acrylate at 4.2. Below this pK_a_ value, the carboxylic groups of the PA hydrogel are protonated and due to attractive forces the hydrogel is collapsed. With increasing the pH to values above 4.2 the carboxylic groups become deprotonated and negatively charged, which results in repulsion of the ionized groups and swelling of the PA hydrogel. To combine the pH dependent PL sensitivity with the VPT we integrated the CdTe QD PA hydrogel hybrid material into a microfluidic valve seat (Fig. 3a), recently reported by Paschew et al. [16]. To apply a permanent flow, the valve contains a bypass channel and allows changing the buffer and pH near the hybrid material. By changing the pH from pH 10.0 to pH 2.0 the hybrid material undergoes a VPT and fully shrinks. At the same time, the channel is opened, evidenced by a giant increase of flow rate (Fig. 2b) and also microscopically observable (Fig. 3c for pH 10.0 and Fig. 3e for pH 2.0). Simultaneously, this process of acidification and valve opening is monitored by quenching the PL of the CdTe QDs, as the hybrid material shows a pronounced PL at pH 10.0 (Fig. 3d) which is totally quenched at pH 2.0 (Fig. 3f).

In contrast to common fluid flow sensors, here, the observation of a discrete flow rate change OFF/ON is realized by PL quenching ON/OFF and thus exhibiting an in situ optical fluid flow control. That fact offers future chemical information processing due to connecting the chemo-mechanical with chemo-optical characteristics.

## Conclusions

In summary, we designed an optical readout system able to detect changes of the pH value by the immobilization of CdTe QDs in a PA hydrogel. The hydrogel was integrated in microfluidic devices, where it was employed as pH sensor and active switching component. A great advantage is the fact that the detection of the pH value allows the transfer of the chemical information to an optical read-out platform for further post-processing, as the pH value is directly related with the intensity of light signals caused by the emission of the QDs. Of particular importance is the integration of the hybrid material in a well-defined microfluidic sensor chamber that allows investigating the hybrid material in a feasible way by glass fiber spectroscopy. Furthermore, the pH dependent swelling behavior of the polyelectrolyte PA hydrogel was utilized for a microfluidic valve systems that are showing the flow state (OFF or ON) in situ by the tunable PL emission (ON or OFF). With these regards, the PL characteristics of the hybrid material allow beside the optical read-out of the pH value a simultaneous observation of mechanical processes. In the future, the system provides a powerful tool for chemical information processing as it connects chemical information with mechanical and optical properties.

## Abbreviations

Bis: *N*,*N*’-Methylenebis(acrylamide)
EDC: *N*-Ethyl-*N’*-(3-dimethylaminopropyl)carbodiimide
FLIM: Fluorescence lifetime imaging microscopy
Glu: Glutathione
MEMS: Micro electromechanical Systems
NaA: Sodium acrylate
NHS: *N*-Hydroxysuccinimide
NP: Nanoparticle
PA: Poly(acrylate)
PDMS: Poly(dimethylsiloxane)
PET: Poly(ethylene terephthalate)
pH: Negative logarithm of acid concentration
PI: Photoinitiator
pK_a_: Acid dissociation constant
pK_b_: Base dissociation constant
PL: Photoluminescence
QD: Quantum dot
UV: Ultraviolet
VPT: Volume-phase transition
λ_em_: Emission wavelength
λ_ex_: Excitation wavelength
